# Progestins in the symptomatic management of endometriosis: a meta-analysis on their effectiveness and safety

**DOI:** 10.1186/s12905-022-02122-0

**Published:** 2022-12-17

**Authors:** Jon-Benay Mitchell, Sarentha Chetty, Fatima Kathrada

**Affiliations:** 1 Division of Pharmacology, Department of Pharmacy and Pharmacology, Faculty of Health Sciences, University of the Witwatersrand, Johannesburg, South Africa; 2 Division of Clinical Pharmacy, Department of Pharmacy and Pharmacology, Faculty of Health Sciences, University of the Witwatersrand, Johannesburg, South Africa

**Keywords:** Endometriosis, Progestins, Pain relief, Adverse effects

## Abstract

**Background:**

Endometriosis is a complex chronic disease that affects approximately 10% of women of reproductive age worldwide and commonly presents with pelvic pain and infertility.

**Method & outcome measures:**

A systematic review of the literature was carried out using the databases Pubmed, Scopus, Cochrane and ClinicalTrials.gov in women with a confirmed laparoscopic diagnosis of endometriosis receiving progestins to determine a reduction in pain symptoms and the occurrence of adverse effects.

**Results:**

Eighteen studies were included in the meta-analysis. Progestins improved painful symptoms compared to placebo (SMD = −0.61, 95% CI (−0.77, −0.45), *P* < 0.00001) with no comparable differences between the type of progestin. After median study durations of 6–12 months, the median discontinuation rate due to adverse effects was 0.3% (range: 0 − 37.1%) with mild adverse effects reported.

**Conclusion:**

The meta-analysis revealed that pain improvement significantly increased with the use of progestins with low adverse effects.

**Systematic Review Registration:**

PROSPERO CRD42021285026.

**Supplementary Information:**

The online version contains supplementary material available at 10.1186/s12905-022-02122-0.

## Background

Endometriosis is a chronic condition defined by the presence of endometrial-like tissue outside the uterus in the ovaries, the rectovaginal septum, and the pelvic peritoneum [1]. The disease occurs globally and more commonly affects women of reproductive age, causing a significant impact on quality of life [2]. The presentation of endometriosis ranges from lesions within the pelvic cavity to extra pelvic lesions. Endometriotic lesions within the pelvic cavity vary from superficial lesions to deep endometrial lesions which may be accompanied by scarring and adhesions. Extra pelvic endometriotic lesions invade the respiratory tract including the nasal mucosa and lungs, the gastrointestinal tract and abdominal wall, the urinary tract, as well as the diaphragm, pleura, pericardium, inguinal canal, cervix, vagina, vulva, and central nervous system [3].

The pathogenicity of endometriosis includes transplantation of endometrial tissue through retrograde menstruation, coelomic metaplasia of the peritoneal lining, and lymphatic and vascular metastasis particularly in extra pelvic lesions, however, the most widely accepted theory is retrograde menstruation [4]. Steroid hormone-sensitive endometrial cells and tissues are deposited on the peritoneal surfaces, causing an inflammatory response. This reaction has been found to co-occur with adhesions, angiogenesis anatomical (tubal) alterations, fibrosis scarring, and neuronal infiltration, resulting in pain and infertility [1]. In an attempt to identify new biomarkers in endometriosis, a recent study evaluating metabolomics highlighted new insights into the pathophysiology of the disease highlighting changes in the metabolic profile of such patients with increases in *β*-hydroxybutyric acid and glutamine metabolites and a decrease in tryptophan as promising potential biomarkers [5]. Another study outlined new insights into the possible relationship between the host microbiome and endometriosis which could serve as possible targets for preventative and therapeutic therapy if a strong link is confirmed [6].

Endometriosis affects approximately 6–10% of women of reproductive age [7]. Ghiasi, Kulkarni, and Missmer in 2020 [8], conducted a review of the global prevalence of endometriosis between January 1989 and June 2019. Amongst the 28 research papers that reported prevalence, 17 provided prevalence estimations in women with infertility, showing a total prevalence of endometriosis of 27%. Likewise, 11 studies looked at the prevalence of endometriosis in females presenting with persistent pelvic pain, showing a 29% overall prevalence of endometriosis. Twelve studies looked at the prevalence of endometriosis in patients who have had a hysterectomy, ovarian cancer, and tubal sterilization and reported a prevalence of 16%, 10%, and 5% respectively. This study also reported the range prevalence of endometriosis in different geographical regions. In Africa, the prevalence ranged from 0.2 to 48%, and in Australia, the range was 3.4–3.7%. The prevalence estimation ranges were much larger in America, Asia, and Europe, being 0.7–70%, 1–72%, and 0.8–70% respectively. A large limitation of this review is the complexity of endometriosis diagnosis, creating challenges in defining a true population prevalence [8].

A variety of risk factors have been suggested but due to the limited knowledge of the initiation processes of endometriosis and the lack of early detection, a distinct discernment between causation and consequence cannot be made. Women with a short menstrual cycle interval, low body mass index, low parity, early age at menarche and family history of endometriosis have been reported to have an increased risk of developing endometriosis [9, 10]. More recent evidence from a systematic review by Shigesi et al. in 2019 [11], suggests that there is an increased risk of autoimmune diseases among women with endometriosis. The study found that systemic lupus erythematous, Sjogren’s syndrome, autoimmune thyroid disease, coeliac disease, rheumatoid arthritis, multiple sclerosis, inflammatory bowel disease and Addison’s disease increased the risk for developing endometriosis, however, the studies were of low quality [11]. The varying study populations and low diagnostic precision among the various studies create a challenge in quantifying the risk factors associated with endometriosis [9].

There are multiple classification systems of endometriosis that were developed by several professional organisations, mainly based on lesion appearance, pelvic adhesions, and anatomic location of the disease [12]. The revised American Society for Reproductive Medicine (rASRM) classification system is the most widely used worldwide and is based on intraoperative findings. It includes one to four stages to quantify the number of lesions and depth of infiltration (Table 1). The ENZIAN classification system is used as a supplementary tool to the rASRM to provide a morphological classification of deep infiltrating endometriosis [13].

**Table 1:** Classification of endometriosis into one of four stages based on a scoring system [12]

Stages	Score	Characteristics of lesions
I (Minimal)	1–5	Minimal with few superficial implants
II (Mild)	6–15	Mild with deeper implants
III (Moderate)	16–40	Moderate with many deep implants Small cysts on one or both ovaries Filmy adhesions present
IV (Severe)	> 40	Severe with many deep implants Large cysts present on one or both ovaries Dense adhesions present

The gold standard for diagnosis in the past was laparoscopic identification with histological verification, however, transvaginal ultrasonography and magnetic resonance imaging are also currently accepted as the primary diagnostic tools. These methods have the advantage of being less invasive. Other disadvantages associated with laparoscopy are the increased risks and costs associated with surgery and the limited number of skilled specialists in endometriosis excision technique. According to a systematic review conducted in 2019, less invasive tests show promising diagnostic potential, however, further research is required before they can be used in a clinical setting [14]. The average interval between the onset of pain and a definitive diagnosis is 10.4 years [1]. In a study conducted by Bontempo and Mikesell in 2020 [15], it was found that 75.2% of patients reported being misdiagnosed with another physical or mental health problem. Endometriosis remains a serious disease that presents complex diagnostic challenges, including non-specific symptoms, symptom normalisation, lack of awareness amongst the population, and stigma associated with the disease [10].

Pain is one of the most common symptoms of endometriosis, however, this is not an indicator of the severity of the disease and there are a variety of symptoms depending on the location of the endometriomas. Adequate knowledge regarding the signs and symptoms of endometriosis aids in the early detection and diagnosis of the disease [16]. In a qualitative, interview-based study conducted by Fauconnier et al. [16], five classifications of excruciating symptoms due to endometriosis was identified. These include “severe pelvic pain and dysmenorrhoea, dyspareunia, gastrointestinal symptoms, bladder symptoms, such as dysuria, and other symptoms, which included physical and psychological impairment of daily activity, difficulties in daily life and work activities as well as impairment of the participant’s sexual life and their relationship with their partner” (p2689) [16]. These categories consist of distinct symptoms comprising of painful menstruation, paralyzing pain that affects mobility, lower abdominal pain, sharp pain during intercourse, pain on passing stool, bloating, nausea, vomiting, feeling the need to urinate often, extreme exhaustion, dizziness, and fainting as well as pain radiating towards the patient’s breasts or shoulders, amongst many others. The symptoms reported by the participants to be the most ‘severe’, ‘incapacitating’, and ‘getting worse with time’ was pelvic pain and dysmenorrhoea [16].

Management of endometriosis includes both pharmacological therapy and/or surgical intervention aimed at symptomatic relief of pain, improving quality of life (QoL), delaying recurrence, and preserving fertility. The choice of therapy depends on patient factors, cost, extent and location of disease, patient preferences, and previous treatment. Medications indicated for the management of endometriosis include either hormonal therapy (combined oral contraceptives, GnRH agonists, aromatase inhibitors, and danazol) or pain therapy (NSAIDs) with hormonal therapy not indicated in women who wish to conceive [1]. Surgical excision of endometriomas improves fertility however expert technique is required to reduce the risk of complications and morbidity associated with surgery, particularly in deep infiltrating endometriosis [17]. Among the hormonal therapy indicated in the treatment of endometriosis are progestins. Progesterone is a steroid hormone predominantly produced in the ovaries, adrenal glands, and placenta. This hormone has an important role in inhibiting the proliferation of the endometrium and stimulating tissue remodelling until gestation or menstruation. Progestin is a synthetic form of progesterone designed to imitate its action. Progestins were reported to have decreased or eradicated painful symptoms in roughly 90% of patients with endometriosis [7]. Different forms of progestins are widely used in the treatment of endometriosis and are available in different dosages. They are available in the following dosage forms: oral, injectable, intrauterine devices, transdermal patches, vaginal rings as well as subcutaneous implants [18].

The fundamental mechanism by which endometriosis occurs is still unknown, therefore, the exact mechanism whereby progestins control pain is unknown. There are, however, three main mechanisms proposed and these include: (1) the result of active haemorrhaging from endometriotic abrasions; (2) the overexpression of growth factors and pro-inflammatory cytokines, and (3) the inflammation or direct attack of pelvic nerves. Progestin has inhibitory effects on growth factors and angiogenesis, anti-inflammatory actions as well as the ability to induce anovulation. Progestins inhibit gonadotropin-releasing hormone, which in turn suppresses follicular-stimulating hormone and luteinizing hormone secretion [7]. As mentioned above, endometriosis occurs in the presence of oestrogen, however, when progestins are present, it prevents oestrogen-dependent proliferation of the endometrium [19].

The primary progestins used in the symptomatic treatment of endometriosis include dienogest, medroxyprogesterone acetate, norethisterone, and cyproterone which are oral forms of the drug that have been found to have an effective role in decreasing endometriosis-related pelvic pain, controlling excessive uterine bleeding, and improving patient quality of life. Depot medroxyprogesterone acetate and the levonorgestrel intrauterine system are effective in the suppression of endometriosis-related symptoms [7]. A new approach to pain relief in endometriosis patients is the etonogestrel subdermal implant. This drug is well-tolerated and safe to use [11]. Bloating, weight gain, hot flushes, acne, loss of libido and fatigue are commonly reported side effects of progestins with less common reports of mood swings and depression [7].

Endometriosis is a chronic disease that is under-researched, under-reported, and underdiagnosed [20]. A qualitative descriptive study that was conducted using focus group discussions in Australia conducted by Moradi et al. provides comprehensive experiences of women that are living with endometriosis [20]. All the women in this study suffered from severe and progressive pain both cyclical and non-cyclical and presented with other non-specific symptoms such as exhaustion, diarrhoea, and sleep disturbances. The study participants reported that the medical experts arbitrarily dismissed their symptoms as insignificant due to their non-specific nature and link to the menstrual cycle [20]. The debilitating nature of the disease has a significant impact on the psychological well-being of the patient, leading to absenteeism from school or work and overall poor QoL [20]. Even though endometriosis impacts patient QoL, recent studies have found that hormonal treatment, especially progestins compared to combined oral contraceptives, significantly improved participants’ QoL and led to a greater reduction in endometriotic lesions and pain symptoms [21]. However, recurrence of pain symptoms, regardless of the type of hormonal therapy, following cessation of therapy is common and chronic therapy is required [17].

The treatment of endometriosis-associated pain focuses on systemic or local oestrogen suppression, inhibition of tissue proliferation and inflammation or both [22]. Due to the complexity of the disease, treatment needs to be modified and personalised based on the disease severity, symptoms as well as fertility goals of each patient. Some goals of treatment include egg preservation, hormonal suppression, decreased recurrence, limiting the amount of deformation of anatomical structures when performing surgery, as well as psychotherapy [22]. Endometriosis therapy requires a multimodal approach from experienced experts including experts in other organ systems that may be affected [10].

Given the high burden, high prevalence, multi-faceted impact and rapid progression of the disease, increased attempts at improved awareness and education regarding endometriosis require emphasis to ensure early detection and intervention. Furthermore, training medical professionals in the individualised therapeutic approach of endometriosis taking all patient factors into account are imperative for positive outcomes [23].

## Methods

### Literature search

Using the Preferred Reporting Items for Systematic Reviews and Meta-Analyses (PRISMA) guidelines [24], a review was conducted on the efficacy and safety of progestins. Four databases, Pubmed, Scopus, Cochrane and ClinicalTrials.gov were searched for the relevant published literature - on clinical trials and observational studies published between 1 January 2011 and 1 January 2021 using the following MeSH terms: “endometriosis”, “progestins”, “pain relief” and “adverse effects.” The results were compiled into a Mendeley Reference Manager v1.19.6 library [25].

Relevant articles were identified using PICO (population, intervention, comparison, outcome) as a starting point for inclusion criteria.

P - Woman with a validated diagnosis of endometriosis.

I - Progestins

C- Placebo/No treatment

O - Primary outcome measure was pain improvement/reduction in pain intensity.

Secondary outcome measure was the occurrence of adverse effects.

Inclusion criteria: Studies with women with a validated diagnosis of endometriosis, and treatment with progestins were included. Observational (cohort studies) and experimental (randomised controlled and uncontrolled trials) studies were included in the study. The search was restricted to research articles originally written in English.

Exclusion criteria: Non-human (animal & in-vitro) studies, case series and case reports were excluded. Studies that did not measure pain improvement as an outcome measure, included patients with extra pelvic endometriosis, and studies with asymptomatic participants were excluded.

Screening of articles retrieved was independently carried out by two reviewers (JM and YP), following a two-step process. The first step was the screening of the titles and abstracts, followed by full-text screening using the PICO statement and predefined inclusion and exclusion criteria. Discrepancies were resolved by discussion or bringing in a third reviewer (SC or FK).

**Fig. 1:**
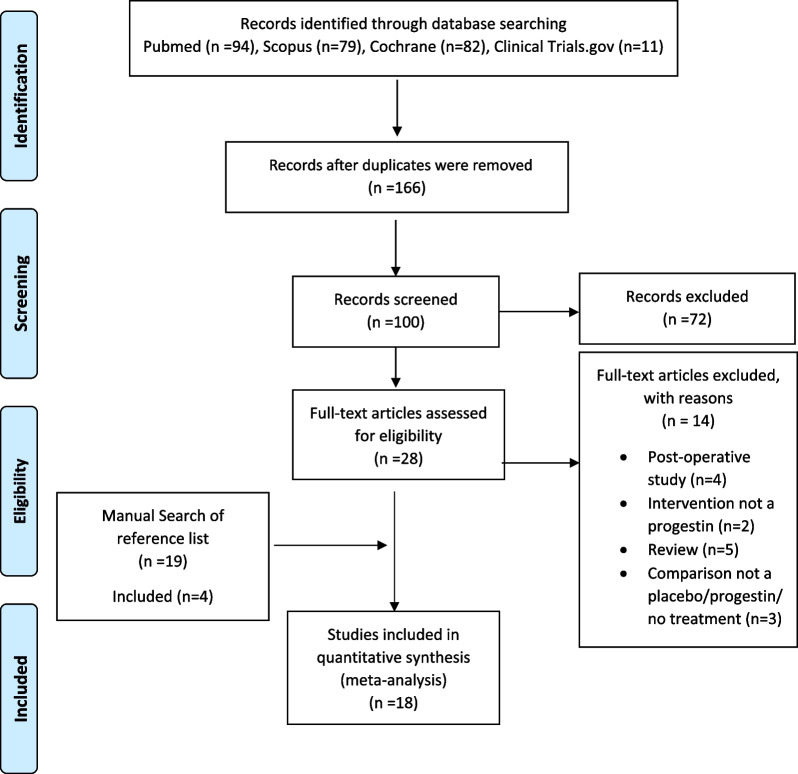
The PRISMA flow diagram, outlining the organization of the selection process through the different steps of the systematic review [26]

### Quality evaluation

The CASP (Critical Appraisal Skill Program), due to its suitability and usability in cohort study types, was used to appraise the quality of the observational studies [27]. This appraisal tool consisted of 12 questions that covered three main issues in an observational study: (1) Are the results of the study valid?; (2) What are the results? and (3) Will the results help locally? Most questions required a ‘yes’, ‘no’ or ‘can’t tell’ as an answer, while others required more detailed explanations.

**Fig. 2:**
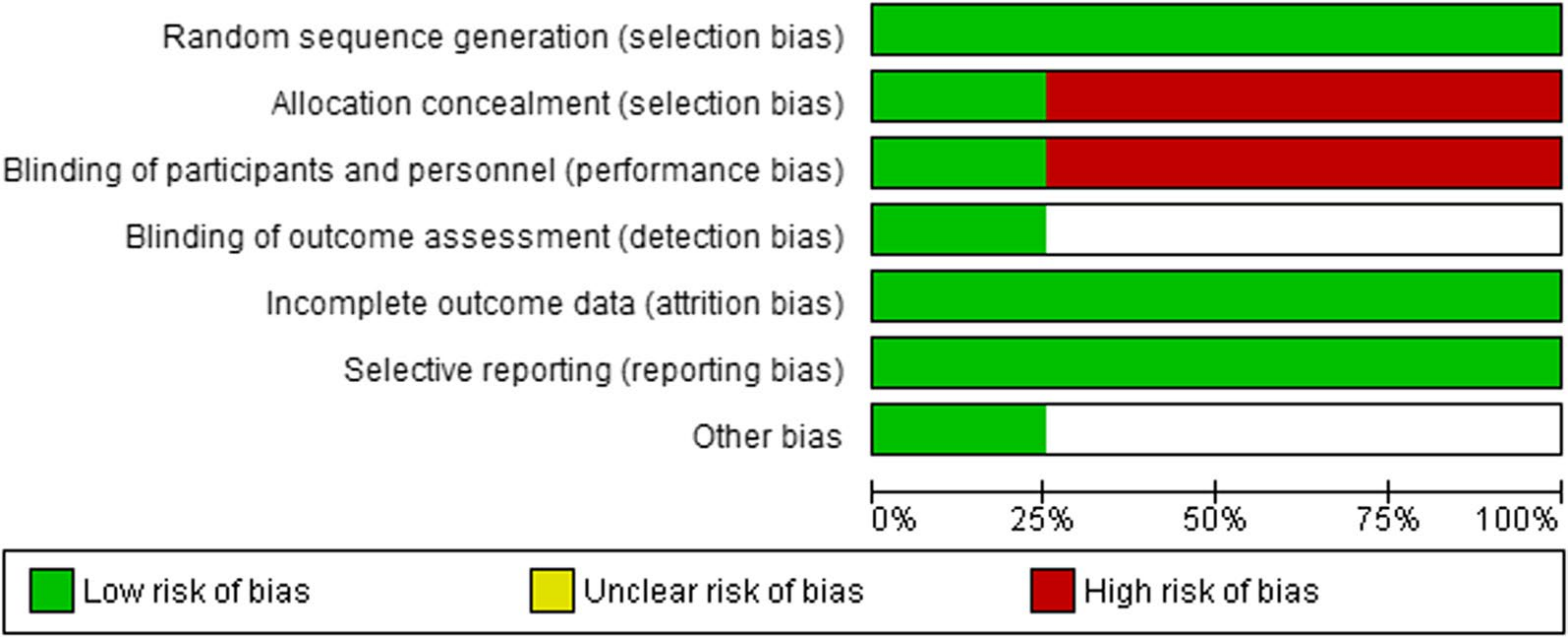
Risk of bias graph: a review of authors’ judgements about each risk of bias item presented as percentages across all RCTs.

The RoB 2.0 Cochrane Risk of Bias Tool for Randomised Trials was used to appraise randomised controlled trials (RCTs) [28]. This assessment consisted of 5 domains with 3–7 questions each requiring a ‘yes’ or ‘no’ answer. At the end of each domain, the bias is rated using judgement (high, low, unclear) based on domains (selection, performance, attrition, reporting and others). The results are summarized graphically in Fig. 2 [28].

The final systemic review was subjected to the PRISMA Checklist Tool [24] to ensure inclusion of all components for a systematic review (Additional file 1)..

### Data extraction

The following information was extracted from the articles included: authors, publication year, study design, number of participants, interventions, comparisons, and study outcomes (pain improvement and adverse effects).

### Statistics

Descriptive statistics were used to summarise study characteristics. *Review Manager (RevMan) Version 5.4.1*, [29] was used to perform the meta-analysis of the two-arm studies. For the single-arm studies, the meta-analysis was carried out using Comprehensive Meta-Analysis (CMA) Version 3.3.070 [30] and the forest plot was created using *Stata Statistical Software Release 17* [31]. All funnel plots were created in RevMan.

Relative risk was used as the summary statistic for dichotomous data and mean difference (MD), or standardised mean difference (SMD) was used for the continuous data. Standardised mean difference, 95% confidence intervals (CI), MD, effect size and heterogeneity were calculated using both the RevMan and CMA software. The I² was used to determine heterogeneity. A fixed-effect model was used when it was hypothesized that the effect would be similar in every study; otherwise, a random-effect model was used. The random-effect model was useful when comparing many studies to estimate the distribution of effects. The random-effect model was used for the analysis of the single-arm studies and the studies reporting adverse effects, while a fixed-effect model was used when analysing the studies comparing progestins and placebos as well as studies in which an etonogestrel-releasing implant is compared to a levonorgestrel-releasing intrauterine system. Statistical significance was attributed to *P*-values less than 0.05.

In the study conducted by Margatho et al. [32] in 2020 the results were presented as Mean ± SE and the following formula, found in the Cochrane Handbook for Systematic Reviews of Interventions, was used to calculate the SD (Eq. 1).

$$SD=SE \times \sqrt{N}$$

Equation 1: Calculation to determine the standard deviation (SD) from reported standard error (SE) [33].

## Results

### Article inclusion

In the initial search, 266 relevant articles were retrieved, with 166 articles remaining following the removal of duplicates. After reviewing the titles and abstracts, 138 articles were excluded. Of the 28 full-text articles reviewed, 14 were excluded. The most common reasons for exclusion were that the studies were done post-operatively (*n* = 4), they compared the efficacy and safety of progestins to other endometriosis treatment options such as gonadotropin-releasing hormone agonists and combined oral contraceptives (*n* = 3), and some of the studies were reviews (*n* = 5). A manual search of the reference lists of the full-text articles was conducted and 19 articles were further retrieved and reviewed. Of these, 4 studies met the inclusion criteria. This process resulted in a total of 18 articles included for analysis (Fig. 1).

Table 2 summarises the study designs, participants, progestins used and study outcomes of the final 18 studies included in this systematic review. Four studies were RCTs and 14 were observational studies.

**Table 2:** Characteristics of the studies on the use of progestins for the symptomatic relief of endometriosis pain

Source	Study design	Year of enrolment	Description of main symptoms	Number of patients enrolled	Study drug	Comparator	Number of patients treated with the study drug	Number of patients treated with comparator	Treatment period	Study outcomes
Petraglia et al. [34]	Randomised open-label, placebo-controlled trial	2004–2007	Pelvic Pain	168	Dienogest 2 mg/d	Placebo	87	81	52 weeks	Pain improvement (VAS) Adverse effects
Morotti et al. [35]	Open-label prospective trial	2014	Pelvic Pain	25	Norethisterone acetate 2.5-5 mg/d	Dienogest 2 mg/d	25	25	6 months	Adverse effects
Vercellini et al. [36]	Retrospective and prospective study	2012–2014	Pelvic Pain	180	Norethisterone acetate 2.5	Dienogest 2 mg/d	90	90	6 months	Pain improvement (NRS)
Morotti et al. [37]	Retrospective study	2004–2016	Pelvic Pain	103	Norethisterone acetate 2.5-5 mg/d	N/A	103	N/A	60 months	Pain improvement (VAS) Adverse effects
Maiorana et al. [38]	Observational, single-centre cohort study	2013–2014	Pelvic Pain	132	Dienogest 2 mg/d	N/A	132	N/A	12 months	Pain improvement (VAS) Adverse effects
Römer [39]	Retrospective study	2016	Pelvic Pain	37	Dienogest 2 mg/d	N/A	37	N/A	60 months	Adverse effects
Vercellini et al. [40]	Prospective study	2014	Pelvic Pain	153	Norethisterone 2.5 mg/d	N/A	153	N/A	12 months	Pain improvement (NRS) Adverse effects
Sansone et al. [41]	Multicenter prospective observational study	2016	Pelvic Pain	25	Etonogestrel implant	N/A	25	N/A	12 months	Pain improvement (VAS) Adverse effects
Lang et al. [42]	Randomised double-blind, placebo-controlled trial	2013	Pelvic Pain	255	Dienogest 2 mg/d	Placebo	126	129	24 weeks	Pain improvement (VAS) Adverse effects.
Yu et al. [43]	Open-label extension study	2018	Pelvic Pain	220	Dienogest 2 mg/d	Placebo	111	109	28 weeks	Pain improvement (VAS) Adverse effects
Carvalho et al. [44]	Open-label parallel-group, non-inferiority, randomised clinical trial	2016	Pelvic Pain	103	Etonogestrel implant	52 mg Levonorgestrel-releasing intrauterine system	52	51	6 months	Pain improvement (VAS)
Del Forno et al. [45]	Retrospective study	2015	Pelvic Pain	135	Dienogest 2 mg/d	Norethisterone acetate 2.5 mg/d	69	66	12 months	Adverse effects
Ferrero et al. [46]	Retrospective study	2019	Pelvic Pain	44	Etonogestrel implant	N/A	44	N/A	24 months	Pain improvement (VAS) Adverse effects
Cho et al. [47]	Prospective cohort study	2011–2017	Pelvic Pain	3356	Dienogest 2 mg/d	N/A	3356	N/A	12 months	Pain improvement (VAS) Adverse effects
Barra et al. [48]	Retrospective study	2019	Pelvic Pain	83	Dienogest 2 mg/d	N/A	83	N/A	36 months	Pain improvement (VAS) Adverse effects
Margatho et al. [32]	Randomised control trial	2016–2019	Pelvic Pain and dysmenorrhoea	103	Etonogestrel implant	52 mg Levonorgestrel-releasing intrauterine system	52	51	24 months	Pain improvement (VAS)
Nirgianakis et al. [49]	Retrospective cohort study	2017–2018	Pelvic Pain	130	Dienogest 2 mg/d	N/A	130	N/A	36 weeks	Pain improvement (VAS) Adverse effects
Kitawaki et al. [50]	Post-marketing observational study	2016–2017	Pelvic Pain	59	Dydrogesterone 10 mg twice daily	N/A	59	N/A	six 21-day cycles	Pain improvement (VAS) Adverse effects

*VAS* Visual analogue scale, *NRS* Numerical rating scale

### Publication bias

The Cochrane Collaboration’s risk of bias tool [17] was used to conduct a quality assessment of the RCTs (*n* = 4) reviewed in this study. The risk of bias graph (Fig. 2) was created using RevMan.

### Effect of progestin on pain improvement

**Fig. 3:**
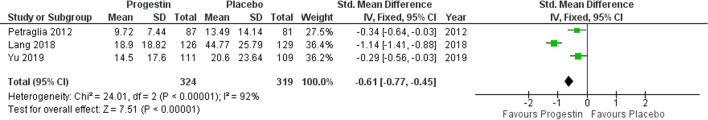
Forest plot of studies in which dienogest (2 mg/d) is compared to a placebo using the VAS to evaluate chronic pelvic pain

**Fig. 4:**
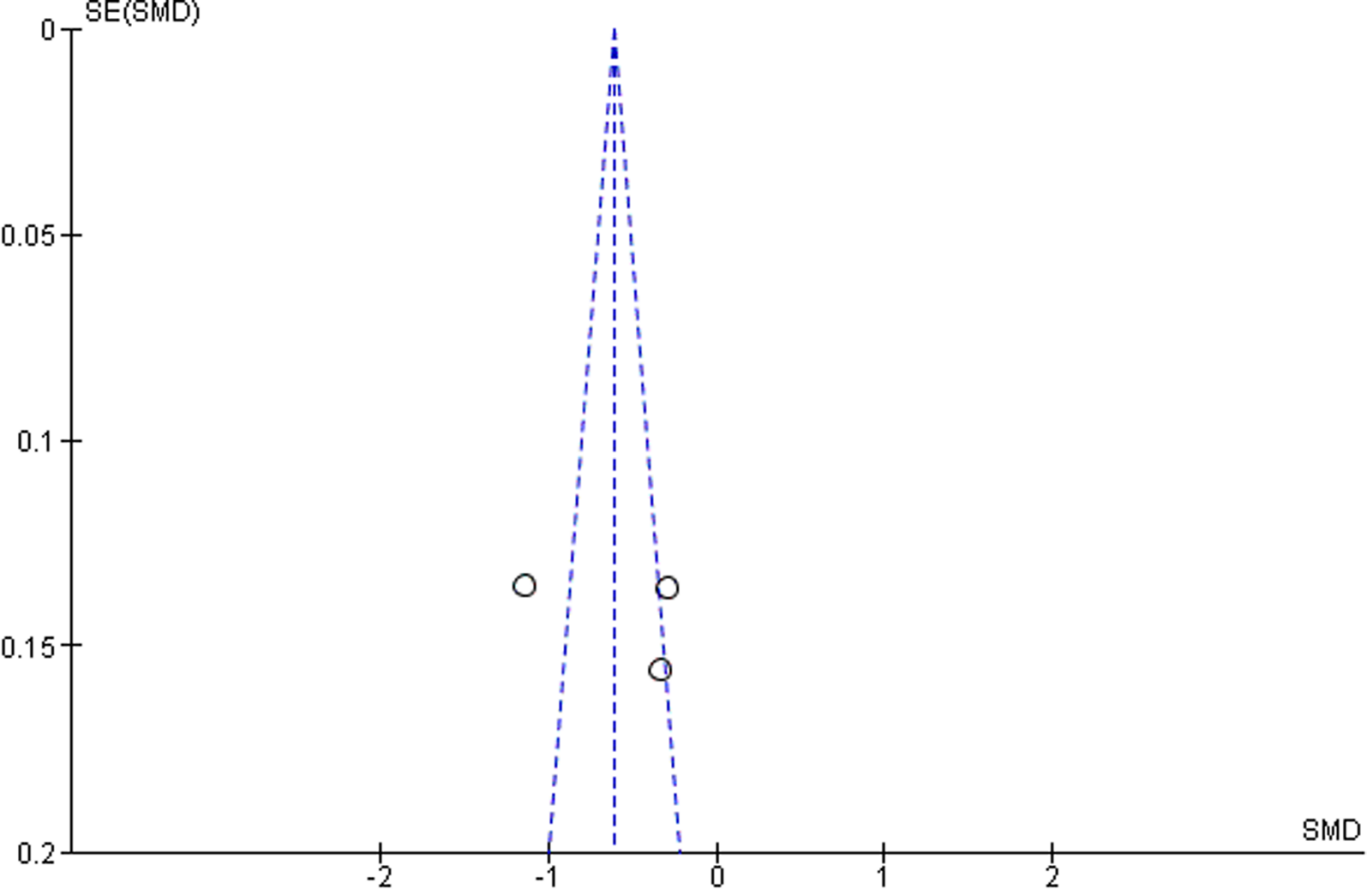
Funnel plot for a meta-analysis of the studies in which dienogest is compared to a placebo

In 12 articles, the visual analogue scale (VAS) was used to evaluate chronic pelvic pain in women with endometriosis. These articles were separated into 2 groups, studies in which progestins were compared to a placebo (*n* = 3) or a different progestin (*n* = 2) (Fig. 3), and studies which included no comparator (*n* = 7) (Fig. 4). The results were analysed to determine the efficacy of progestins in reducing pelvic pain.

As seen in Fig. 3, progestins showed a statistically significant effect in improving painful symptoms caused by endometriosis (SMD=−0.61, 95% CI (−0.77, −0.45), *P* < 0.00001). The *I*² value of 92% suggests very high heterogeneity.

The asymmetry of the funnel plot for studies which included a comparator (Fig. 4) is attributed to the high heterogeneity of the studies.

**Fig. 5:**
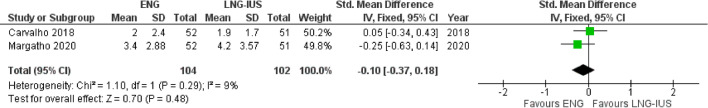
Studies in which an etonogestrel-releasing implant is compared to a levonorgestrel-releasing intrauterine system

Figure 5 shows that there was no significant difference between the etonogestrel-releasing implant and the levonorgestrel-releasing intrauterine system (SMD=−0.10, 95% CI (−0.37, 0.18), *P* = 0.48) in providing pain relief.

**Fig. 6:**
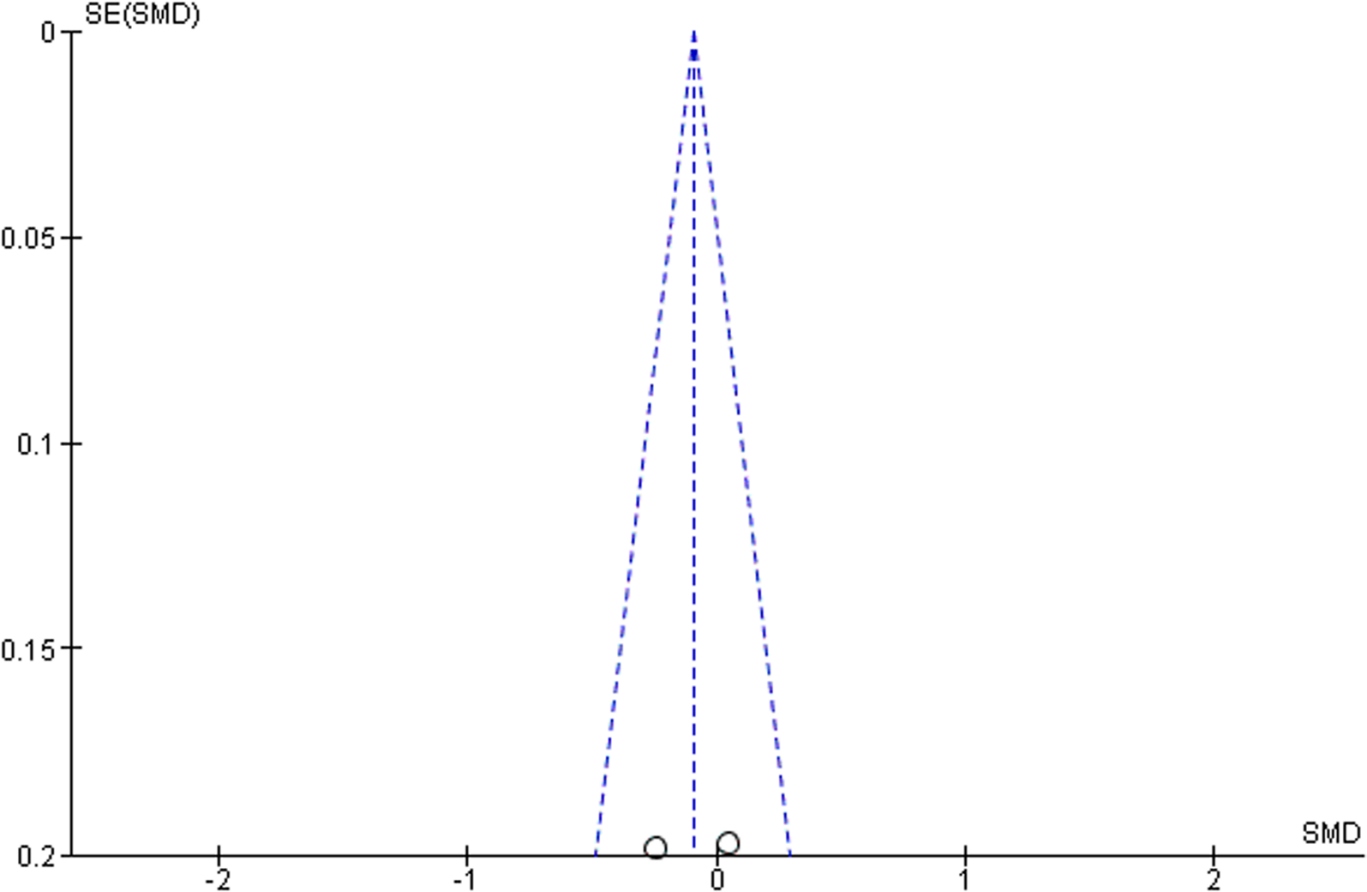
Funnel plot for meta-analysis of studies in which an etonogestrel-releasing implant is compared to a levonorgestrel-releasing intrauterine system

The corresponding funnel plot (Fig. 6) is symmetrical showing that there is little to no bias, good methodological quality and low heterogeneity.

**Fig. 7:**
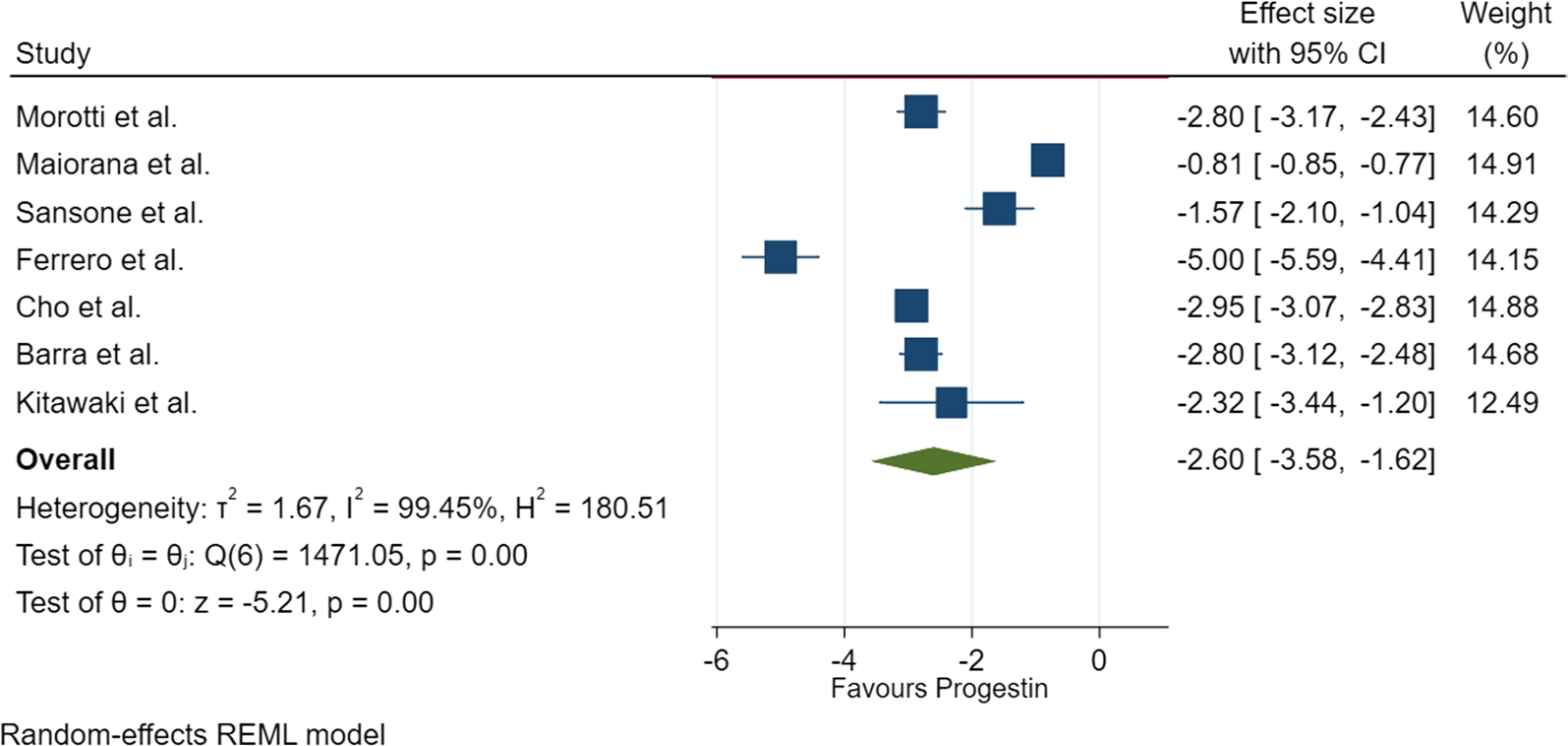
Forest plot for the single-arm studies using VAS to evaluate chronic pelvic pain

As seen in Fig. 7, the total MD is −2.60 with a 95% CI (−3.58, −1.62), proving that progestins are effective in improving chronic pelvic pain caused by endometriosis. The I² value of 99.45% indicates very high heterogeneity and the *P* < 0.00001 suggests that the results are statistically significant.

**Fig. 8:**
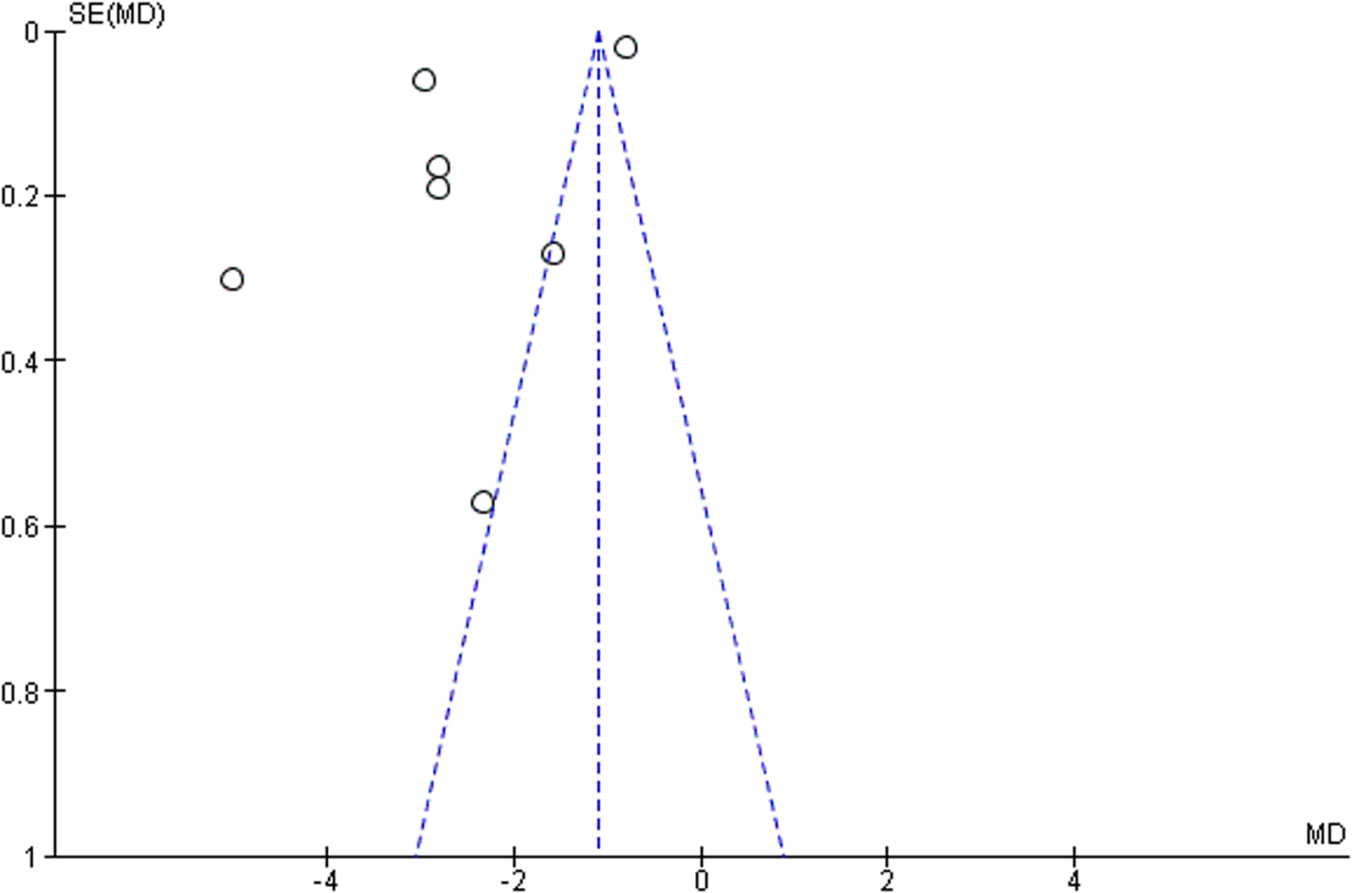
Funnel plot for meta-analysis of the single-arm studies using VAS to evaluate chronic pelvic pain

The asymmetry of the funnel plot in Fig. 8 indicates that there may be some publication bias. The high heterogeneity demonstrates the differences in the interventions and methods used in the various studies.

The studies by Vercellini et al. in 2016 [36] and 2018 [40] measured non-menstrual pelvic pain improvement using a numerical rating scale (NRS). In the 2016 study [36] the severity of pain symptoms caused by endometriosis was assessed by a 0 to 10-point NRS at baseline and after 6 months of treatment in women with endometriosis. In the norethindrone acetate (*n* = 31) and the dienogest (*n* = 29) groups, the median (interquartile range) baseline NRS was 7 (6–7) and decreased to 0 (0–2) after 6 months. The 2018 study [40] produced similar results, where the median (interquartile range) for the baseline NRS was 5 (0–7) and decreased to 0 (0–2) after 12 months in the 125 participants using norethisterone acetate. This indicates that progestins are effective in improving painful symptoms caused by endometriosis.

### Adverse effects caused by progestins

Fifteen of the studies reviewed reported adverse effects. Vercellini et al. 2016 [36] reported adverse effects as a mean and not a percentage and were therefore excluded from the graph.

**Fig. 9:**
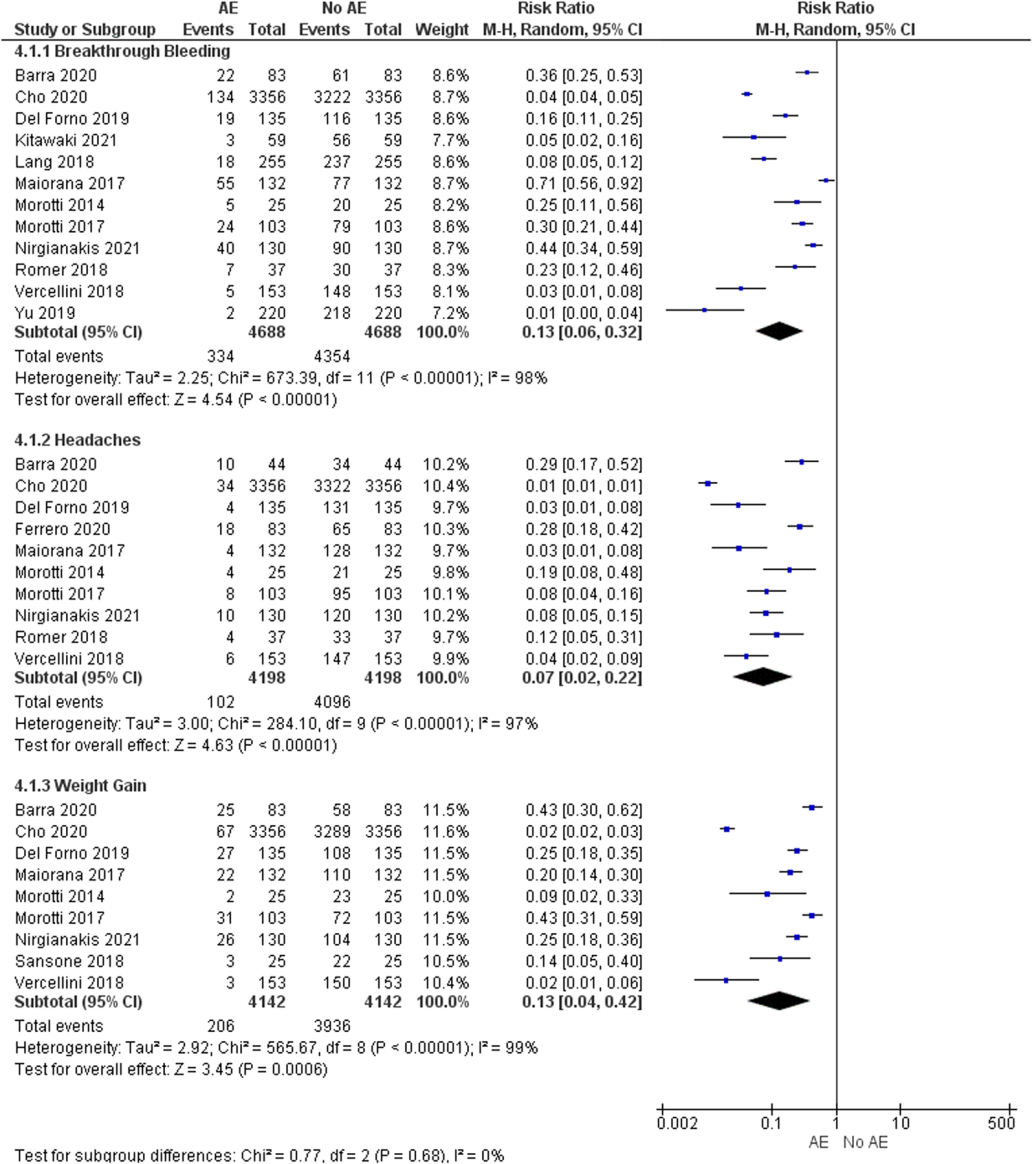
Forest plot showing the adverse effects caused by progestins that occurred most frequently across 14 studies. ‘AE’ represents adverse events and ‘No AE’ represents no adverse events

Figure 9 depicts 3 forest plots for the most common adverse effects that occurred across the remaining 14 studies that reported on adverse effects. All three adverse effects presented considerable heterogeneity and were statistically significant. The subtotal risk ratio (95% CI) for breakthrough bleeding, headaches and weight gain was 0.13 (0.06, 0.32), 0.07 (0.02, 0.22) and 0.13 (0.04, 0.42) respectively, indicating that there is a low risk that progestins will cause the occurrence of an adverse effect.

The complete list of adverse effects and their percentage of occurrence across the 14 studies are presented in a table (Table 3).

**Table 3:** Percentage of adverse effects experienced by participants across the studies reviewed

Source	Year	Number of patients enrolled	Breast discomfort % (*n*)	Nausea % (*n*)	Irritability % (*n*)	Headaches % (*n*)	Breakthrough bleeding % (*n*)	Weight gain % (*n*)	Decreased Libido % (n)	Depression % (*n*)	Vaginal dryness % (*n*)	Acne % (*n*)	Hair Loss % (*n*)
Petraglia et al.	2012	168	4.2 (7)	3 (5)	2.4 (4)								
Morotti et al.	2014	25	4 (1)	8 (2)		16 (4)	20 (5)	8 (2)	4 (1)				
Morotti et al.	2017	103				8.7 (9)	23.3 (24)	30.1 (31)	10.7 (11)	6.8 (7)		4.8 (5)	
Maiorana et al.	2017	132	8.1 (11)			3.6 (5)	42.3 (56)	17.1 (23)	8.1 (11)	16.2 (22)	3.6 (5)		
Römer	2018	37				10.8 (4)	18.9 (7)			10.8 (4)			
Vercellini et al.	2018	153	0.65 (1)	0.65 (1)		4.5 (7)	3.9 (6)	2.6 (4)	0.65 (1)		0.65 (1)	1.3 (2)	
Sansone et al.	2018	25						12.5 (3)					
Lang et al.	2018	255					7.9 (20)						
Yu et al.	2019	220	1.8 (4)			0 (0)	1.8 (4)						
Del Forno et al.	2019	69	1.4 (1)	0 (0)	1.4 (1)	2.9 (2)	8.7 (6)	14.5 (10)	7.2 (5)		7.2 (5)	1.4 (1)	2.9 (2)
		66	7.6 (5)	0 (0)	7.6 (5)	3 (2)	21.2 (14)	30.3 (20)	18.2 (12)		10.6 (7)	1.5 (1)	0
Ferrero et al.	2020	44				23.3 (10)						7 (3)	
Cho et al.	2020	3356	1.25 (42)	0.64 (22)		1.32 (45)	4.18 (141)	2.6 (88)		0.77 (26)		0.77 (26)	0.32 (11)
Barra et al.	2020	83				21.2 (18)	26.9 (23)	30.1 (25)	2.8 (3)	9.6 (8)		1.9 (2)	
Margatho et al.	2020	103											
Nirgianakis et al.	2020	130	15 (20)			8 (11)	31 (40)	20 (26)	19 (25)	33 (43)		10 (13)	6 (8)
Kitawaki et al.	2021	59					5.1 (3)

**Fig. 10:**
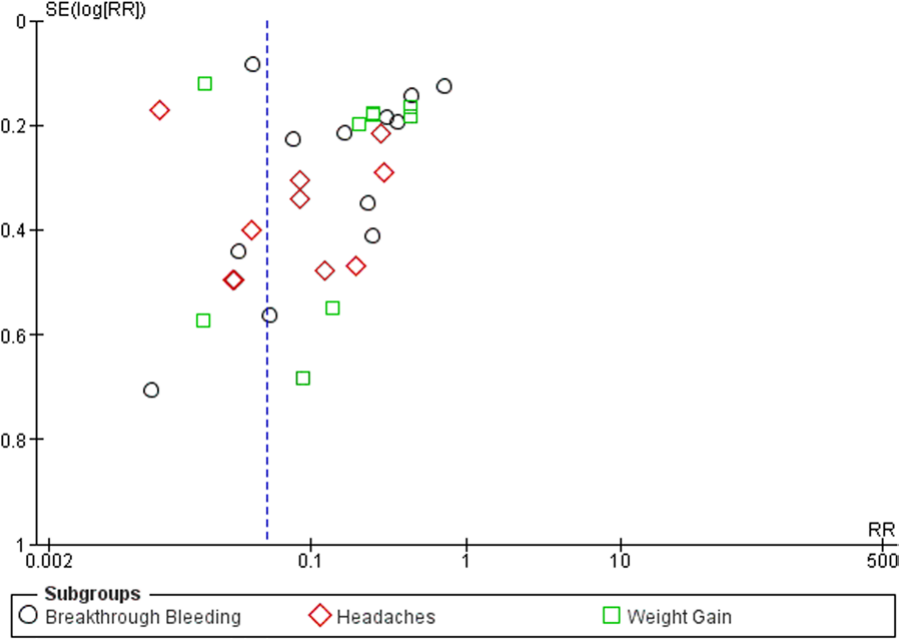
Funnel plot for studies showing the common adverse effects reported by progestins

The funnel plot (Fig. 10) asymmetry is due to the high heterogeneity in each subgroup. Publication bias is an unlikely cause, however, selective outcome reporting and analysis reporting may be a cause for the asymmetry, due to the subjective nature of the responses in each study. It could also be attributed to the varying methods used in each study.

## Discussion

Among the approximate 10% of women presenting with endometriosis globally, about 80% experience chronic pelvic pain [51]. In a condition with no cure, improvement of pain is essential in improving disease outcomes and quality of life. Therapies with progestins are a more reliable option compared to the usually sought after NSAIDs which do not alter the disease course and are not ideal for long-term use due to the adverse gastrointestinal effects. Progestins, on the other hand, display oestrogen suppressive effects useful in decreasing the growth of endometrial tissue and possess anti-inflammatory, anti-proliferative and anti-angiogenic effects applicable in the mechanisms associated with the course of disease in endometriosis. Based on the existing literature, this study reveals that progestins are an effective symptomatic therapy in patients with endometriosis.

In the 3 studies comparing progestins to placebos, the use of progestins was associated with a positive reduction in pelvic pain in women with endometriosis. All 3 studies had a similar design with the use of 2 mg/day of dienogest reporting pain outcomes using the same VAS scale. All 3 studies also reported a favourable safety and tolerability profile, and a low rate of treatment-related discontinuations over periods of up to 52 weeks. Although the dienogest displayed de-synchronous uterine bleeding effects in the short-term, the frequency and intensity of menstrual bleeding were reduced with long-term use (24–52 weeks), however, bleeding frequency and intensity returned on discontinuation of the drug, indicating the need for long-term use for a sustained beneficial effect. The studies done by Lang et al. (2018) [42] and Yu et al. (2019) [43] were conducted in a Chinese population, while the study done by Petraglia et al. (2011) [34] was done on women in Germany, Italy, and Ukraine, indicating that the effects apply to both Caucasian and Chinese populations. This highlights the need for further similar studies in other population groups to ascertain if these positive outcomes apply to all population groups.

In a comparison between non-oral progestins, there was no significant difference between the pain-relieving effects of the etonorgestrel subdermal implant compared to the levonorgestrel intrauterine system (LNG-IUS), with both producing similar pain-relieving effects [32, 44]. The implant and the intrauterine device provide the option of convenience in removing the daily pill burden, however, as seen in both studies, there was a large discontinuation rate, many of which were due to uterine bleeding irregularities which highlight the need for larger scale studies in the long-term acceptability of these alternatives to oral progestins.

In the study by Vercellini et al. (2016), the non-menstrual pelvic pain associated with endometriosis was diminished in both the norethindrone acetate (pre-study) and the dienogest (post study) groups, however, there was no significant ameliorations in pain relief following the introduction of dienogest [36].

The findings among the single-arm studies were consistent in showing a beneficial effect of progestins in providing pain relief in patients with endometriosis (P < 0.00001). Of the 7 studies included in this analysis, 4 used dienogest, 2 used norethindrone acetate, 2 used the etonogestrel implant and 1 used dydrogesterone. All four of these progestins significantly reduced the severity of non-menstrual pelvic pain experienced by women with endometriosis and the adverse effects related to these drugs was minimal and mild in nature. It must be noted however that these studies lacked a placebo or comparator group.

One of the most common adverse effects that occurred due to progestin use across the studies was weight gain, with 2 studies on norethindrone acetate, Morotti et al. (2017) [37] and Del Forno et al. (2019) [45], and 1 study on dienogest by Barra et al. [48] reporting a 30% occurrence. Breakthrough bleeding, another common adverse effect occurred in 13 out of the 14 studies reviewed on reported adverse effects.

The majority of the studies included reported on oral progestins, particularly dienogest and/or norethisterone with a limited number of studies reporting on subdermal implants and/or the LNG-IUS. There was homogeneity in the VAS score pain management tool used across the majority of the studies, however, the treatment periods and sample sizes varied from 24 weeks to 5 years and 25 to 3356 respectively.

In addition to pelvic pain relief, many of the studies provided consistent reports on the effects of progestins in relieving dyspareunia which ultimately plays a major role in the quality of life in patients with endometriosis. The studies included patients with all stages of endometriosis with no specific correlations being made in progestin responses between the mild stages (I, II) and advanced stages (III, IV) of endometriosis to identify phenotypes with possible poor response to progestins. Further large studies aimed specifically at the staging and response to progestins would be beneficial. No clear distinguishable differences can be drawn from the current studies concerning the effects of the treatment with progestins alone in comparison to the use of progestins following surgery to inform clinical practice guidelines.

The high heterogeneity in this review can be attributed to the differences between the methods of the included studies. This review included observational studies and randomised controlled trials which additionally contributed to the high percentage of heterogeneity. The varied sample sizes and progestin formulations across the studies contributed to the variability of the results with the single-arm studies lacking a comparator and varying study designs as clear contributors to heterogeneity.

### Limitations

The main limitation of this systematic review is the small number of studies that met the strict inclusion criteria. Another limitation is that a few studies were open-label randomised control trials that did not use blinding and/or allocation concealment, which increases the risk of potential bias. The CASP tool used to appraise the quality of the observational studies revealed that there is potential bias when measuring the outcomes in some studies because occasionally questionnaires were used, and this is not always an accurate measure of outcomes. In the progestin vs. placebo studies, the decrease in pain for the progestin arm was informative, however, the decrease in pain measurements from baseline in the placebo arm can potentially be misleading due to the placebo effect. Another limitation is selection bias, where study participants could potentially have less severe forms of endometriosis, which would make the study less generalisable. This study only evaluates the effect progestins have on chronic pain from the start of treatment until the end of treatment and does not take into consideration the effects of discontinuing treatment, and whether chronic pain recurs once treatment has ceased. The results of this study should be considered with caution and expanded on in future studies.

Future studies should consider the efficacy and safety of the long-term use of progestins. Only 2 studies in this review had a duration of up to 5 years and endometriosis is a chronic disease that requires long-term treatment. Five different types of progestins were reviewed in this study, and the effects of the individual types and dosages should be studied in the future.

## Conclusion

There is no known cure for endometriosis [52], which has psychological, physical and social effects impacting social activity engagement, fertility, productivity in the workplace, sexual practices and mental health. A demand for more research and awareness regarding treatment effectiveness worldwide is of growing importance to ensure early diagnosis as well as effective and improved management of the disease and its debilitating symptoms. In a systematic review conducted by D’Alterio et al., it was found that medical and surgical interventions for endometriosis improved patients’ quality of life, however, recurrence is frequent [23]. This study demonstrated that progestins produced positive pain relief and improvements in quality of life in patients with endometriosis. However, treatment needs to be personalised taking into account the goals of the patient, surgical candidacy, fertility planning and patient preference [23].

This review of 18 studies concluded that progestins prove to be a safe and effective treatment for chronic pelvic pain in women with endometriosis, with a good tolerability profile.

## Supplementary Information

**Additional file 1.** PRISMA Checklist Tool.

## Data Availability

The datasets used and/or analysed during the current study is available from the corresponding author on reasonable request.

## Abbreviations

BMI: Body mass index
QoL: Quality of life
NSAID: Non-steroidal anti-inflammatory drugs
GnRH: Gonadotropin-releasing hormone
PRISMA: Preferred reporting items for systematic reviews and meta-analyses
MeSH: Medical subject headings
PICO: Patient/population, intervention, comparison, and outcomes
RCT: Randomised control trials
CASP: Critical appraisal skills programme
RevMan: Review manager
CMA: Comprehensive meta-analysis
MD: Mean difference
SMD: Standardised mean difference
CI: Confidence interval
VAS: Visual analogue scale
NRS: Numerical rating scale

## References

[1] Giudice LC, Kao LC (2004). Endometriosis. The Lancet.

[2] Pospisilova E, Kiss I, Souckova H, Tomes P, Spicka J, Matkowski R (2019). Circulating endometrial cells: a new source of information on endometriosis dynamics. J Clinic Med.

[3] Machairiotis N, Stylianaki A, Dryllis G, Zarogoulidis P, Kouroutou P, Tsiamis N (2013). Extrapelvic endometriosis: a rare entity or an underdiagnosed condition?. Diagn Pathol.

[4] Sasson IE, Taylor HS (2008). Stem cells and the pathogenesis of endometriosis. Annals  New York Acad Sci.

[5] Murgia F, Angioni S, D’Alterio MN, Pirarba S, Noto A, Santoru ML (2020). Metabolic profile of patients with severe endometriosis: a prospective experimental study. Reproduc Sci.

[6] D’Alterio MN, Giuliani C, Scicchitano F, Langana AS, Oltolina NM, Sorrentino F (2021). Possible role of microbiome in the pathogenesis of endometriosis. Minerva Obstet Gynecol.

[7] Gezer A, Oral E (2015). Progestin therapy in endometriosis. Women’s Health.

[8] Ghiasi M, Kulkarni MT, Missmer SA (2020). Is endometriosis more common and more severe than it was 30 years ago?. J Minim Invasive Gynecol.

[9] Peterson CM, Johnstone EB, Hammoud AO, Stanford JB, Varner MW, Kennedy A (2013). Risk factors associated with endometriosis: importance of study population for characterizing disease in the ENDO Study. Am J Obstet Gynecol.

[10] Zondervan KT, Becker CM, Missmer SA (2020). Endometriosis. New England J Med.

[11] Shigesi N, Kvaskoff M, Kirtley S, Feng Q, Fang H, Knight JC (2019). The association between endometriosis and autoimmune diseases: a systematic review and meta-analysis. Human Reproduct Update.

[12] Lee S-Y, Koo Y-J, Lee D-H (2021). Classification of endometriosis. Yeungnam Univ J Med.

[13] Johnson NP, Hummelshoj L, Adamson GD, Keckstein J, Taylor HS, Abrao MS (2016). World endometriosis society consensus on the classification of endometriosis. Human Reproduc.

[14] Kiesel L, Sourouni M (2019). Diagnosis of endometriosis in the 21st century. Climacteric.

[15] Bontempo AC, Mikesell L (2020). Patient perceptions of misdiagnosis of endometriosis: results from an online national survey. Diagnosis.

[16] Fauconnier A, Staraci S, Huchon C, Roman H, Panel P, Descamps P (2013). Comparison of patient- and physician-based descriptions of symptoms of endometriosis: a qualitative study. Hum Reprod.

[17] Becker CM, Gattrell WT, Gude K, Singh SS (2017). Reevaluating response and failure of medical treatment of endometriosis: a systematic review. Fertil Steril.

[18] Gheorghisan-Galateanu A (2019). Hormonal therapy in women of reproductive age with endometriosis: an update. Acta Endocrinol (Bucharest).

[19] Vercellini P, Buggio L, Berlanda N, Barbara G, Somigliana E, Bosari S (2016). Estrogen-progestins and progestins for the management of endometriosis. Fertility  Sterility.

[20] Moradi M, Parker M, Sneddon A, Lopez V, Ellwood D (2014). Impact of endometriosis on women’s lives: a qualitative study. BMC Women’s Health.

[21] Piacenti I, Viscardi MF, Masciullo L, Sangiuliano C, Scaramuzzino S, Piccioni MG (2021). Dienogest versus continuous oral levonorgestrel/EE in patients with endometriosis: what’s the best choice?. Gynecol Endocrinol.

[22] Nezhat C, Vang N, Tanaka PP, Nezhat C (2019). Optimal management of endometriosis and pain. Obstetrics  Gynecol.

[23] D’Alterio MN, Saponara S, Agus M, Laganà AS, Noventa M, Loi ES (2021). Medical and surgical interventions to improve the quality of life for endometriosis patients: a systematic review. Gynecol Surg.

[24] PRISMA [Internet]. PRISMA Checklist; [2020]. Available from: http://prisma-statement.org/PRISMAStatement/Checklist.aspx. Accessed 23 June 2021.

[25] Mendeley [Internet]. Mendeley.com; [2021]. Available from: https://www.mendeley.com/search/. Accessed 9 October 2021.

[26] PRISMA [Internet]. PRISMA Flow Diagram; [2020]. Available from: http://prisma-statement.org/PRISMAStatement/FlowDiagram.aspx. Accessed 23 June 2021.

[27] CASP [Internet]. CASP CHECKLISTS; [2020]. Available from: https://casp-uk.net/casp-tools-checklists/. Accessed 23 June 2021.

[28] Cochrane [Internet]. RoB 2: A revised cochrane risk-of-bias tool for randomized trials; [2011]. Available from: https://methods.cochrane.org/bias/resources/rob-2-revised-cochrane-risk-bias-tool-randomized-trials. Accessed 23 June 2021.

[29] Cochrane [Internet]. RevMan; [2020]. Available from: https://training.cochrane.org/online-learning/core-software-cochrane-reviews/revman. Accessed 1 November 2021.

[30] Meta-analysis.com [Internet]. Version 3 Software | Comprehensive Meta-Analysis; [2021]. Available from: https://www.meta-analysis.com/pages/downloadV3.php?&email=jbmitchell80%40gmail.com&valid=s214682. Accessed 9 Nov 2021.

[31] STATA [Internet]. StataCorp; [2021]. Available from: https://www.stata.com/. Accessed 18 October 2022.

[32] Margatho D, Carvalho NM, Bahamondes L (2020). Endometriosis-associated pain scores and biomarkers in users of the etonogestrel-releasing subdermal implant or the 52-mg levonorgestrel-releasing intrauterine system for up to 24 months. Eur J Contracept Reproduc Health Care.

[33] Cochrane [Internet]. 7.7.3.2 Obtaining standard deviations from standard errors; [2021]. Available from: https://handbook-5-1.cochrane.org/chapter_7/7_7_3_2_obtaining_standard_deviations_from_standard_errors_and.htm. Accessed 1 Nov 2021.

[34] Petraglia F, Hornung D, Seitz C, Faustmann T, Gerlinger C, Luisi S (2011). Reduced pelvic pain in women with endometriosis: efficacy of long-term dienogest treatment. Archiv Gynecol Obstetr.

[35] Morotti M, Sozzi F, Remorgida V, Venturini PL, Ferrero S (2014). Dienogest in women with persistent endometriosis-related pelvic pain during norethisterone acetate treatment. Eur J Obstet Gynecol Reproduc Biol.

[36] Vercellini P, Bracco B, Mosconi P, Roberto A, Alberico D, Dhouha D (2016). Norethindrone acetate or dienogest for the treatment of symptomatic endometriosis: a before and after study. Fertility Sterility.

[37] Morotti M, Venturini PL, Biscaldi E, Racca A, Calanni L, Vellone VG (2017). Efficacy and acceptability of long-term norethindrone acetate for the treatment of rectovaginal endometriosis. Eur J Obstet Gynecol Reproduc Biol.

[38] Maiorana A, Incandela D, Parazzini F, Alio W, Mercurio A, Giambanco L (2017). Efficacy of dienogest in improving pain in women with endometriosis: a 12-month single-center experience. Archives  Gynecol Obstetr.

[39] Römer T (2018). Long-term treatment of endometriosis with dienogest: retrospective analysis of efficacy and safety in clinical practice. Archiv Gynecol Obstetr.

[40] Vercellini P, Ottolini F, Frattaruolo MP, Buggio L, Roberto A, Somigliana E (2018). Is shifting to a progestin worthwhile when estrogen–progestins are inefficacious for endometriosis-associated pain?. Reproduct Sci.

[41] Sansone A, De Rosa N, Giampaolino P, Guida M, Laganà AS, Di Carlo C (2018). Effects of etonogestrel implant on quality of life, sexual function, and pelvic pain in women suffering from endometriosis: results from a multicenter, prospective, observational study. Archiv Gynecol Obstetr.

[42] Lang J, Yu Q, Zhang S, Li H, Gude K, von Ludwig C (2018). Dienogest for treatment of endometriosis in Chinese women: a placebo-controlled, randomized, double-blind phase 3 study. J Women’s Health.

[43] Yu Q, Zhang S, Li H, Wang P, Zvolanek M, Ren X (2019). Dienogest for treatment of endometriosis in women: a 28-week, open-label, extension study. J Women’s Health.

[44] Carvalho N, Margatho D, Cursino K, Benetti-Pinto CL, Bahamondes L (2018). Control of endometriosis-associated pain with etonogestrel-releasing contraceptive implant and 52-mg levonorgestrel-releasing intrauterine system: randomized clinical trial. Fertil Steril.

[45] Del Forno S, Mabrouk M, Arena A, Mattioli G, Giaquinto I, Paradisi R (2019). Dienogest or Norethindrone acetate for the treatment of ovarian endometriomas: can we avoid surgery?. Eur J Obstet Gynecol Reproductive Biology.

[46] Ferrero S, Scala C, Ciccarelli S, Vellone VG, Barra F (2019). Treatment of rectovaginal endometriosis with the etonogestrel-releasing contraceptive implant. Gynaecol Endocrinol.

[47] Cho B, Roh J-W, Park J, Jeong K, Kim T-H, Kim YS (2020). Safety and effectiveness of dienogest (Visanne®) for treatment of endometriosis: a large prospective cohort study. Reproductive Sci.

[48] Barra F, Scala C, Leone Roberti Maggiore U, Ferrero S. Long-term administration of Dienogest for the treatment of pain and intestinal symptoms in patients with rectosigmoid endometriosis. J Clinic Med 2020; 9(1):154. 10.3390/jcm9010154 PMC7019573 31935969

[49] Nirgianakis K, Vaineau C, Agliati L, McKinnon B, Gasparri ML, Mueller MD (2020). Risk factors for non-response and discontinuation of dienogest in endometriosis patients: a cohort study. Acta Obstetricia et Gynecologica Scandinavica.

[50] Kitawaki J, Koga K, Kanzo T, Momoeda M (2021). An assessment of the efficacy and safety of dydrogesterone in women with ovarian endometrioma: an open-label multicenter clinical study. Reproduc Med Biol.

[51] Maddern J, Grundy L, Castro J, Brierley SM. [Internet]. Pain in Endometriosis. Frontiers in Cellular Neuroscience; [2020]. Available from: https://www.ncbi.nlm.nih.gov/pmc/articles/PMC7573391/. Accessed 8 Nov 2021. 10.3389/fncel.2020.590823 PMC7573391 33132854

[52] World health organization. WHO [Internet]. Endometriosis world health organization; [2021]. Available from: https://www.who.int/news-room/fact-sheets/detail/endometriosis#:~text=It%20can%20decrease%20quality%20of,school%20(8%2C9). Accessed 31 May 2021.

